# First cytogenetic information for *Drymoreomys albimaculatus* (Rodentia, Cricetidae), a recently described genus from Brazilian Atlantic Forest

**DOI:** 10.3897/zookeys.303.4873

**Published:** 2013-05-21

**Authors:** Elkin Y. Suárez-Villota, Camilla B. Di-Nizo, Carolina L. Neves, Maria José de Jesus Silva

**Affiliations:** 1 Laboratório de Ecologia e Evolução, Instituto Butantan, São Paulo, Brazil; 2 Laboratório de Biologia da Conservação, Departamento de Ecologia, Universidade Estadual Paulista (UNESP), Rio Claro, SP, Brazil

**Keywords:** Oryzomyini, karyotype, CBG banding, GTG banding, FISH, IRBP, Cyt *b*

## Abstract

The recently described taxon *Drymoreomys albimaculatus* is endemic to the Brazilian Atlantic Forest and its biology and genetics are still poorly known. Herein, we present, for the first time, the karyotype of the species using classical and molecular cytogenetics, which showed 2n=62, FN=62, and interstitial telomeric signals at the sex chromosomes. Nuclear and mitochondrial DNA sequences from the two karyotyped individuals verify the taxonomic identity as the recently described *Drymoreomys albimaculatus* and confirm the relationship of the species with other Oryzomyini. Additionally, external morphological information is provided.

## Introduction

The Atlantic Forest harbors a high diversity of mammals, 20 percent of which are rodents of the subfamily Sigmodontinae (Ribeiro et al. 2009). However, the fauna of this biome is still barely known, such that discovery of new species is still common (De Vivo et al. 2010). Since 1999, 14 new species of sigmodontines were formally described for Atlantic Forest: *Abrawayaomys chebezi* (Pardiñas et al. 2009), *Akodon paranaensis* (Christoff et al. 2000), *Akodon philipmeyersi* (Pardiñas et al. 2005), *Akodon reigi* (González et al. 1999), *Brucepattersonius paradisus*, *Brucepattersonius guarani*, *Brucepattersonius misionensis* (Mares and Braun 2000), *Cerradomys langguthi*, *Cerradomys vivoi* (Percequillo et al. 2008), *Hylaeamys seuanezi* (Weksler et al. 1999), *Juliomys rimofrons* (Oliveira and Bonvicino 2002), *Juliomys ossitenius* (Costa et al. 2007), *Rhipidomys tribei*, and *Rhipidomys itoan* (Costa et al. 2011).

Recently,Percequillo et al. (2011) described *Drymoreomys albimaculatus* as a new monotypic genus, endemic to the Brazilian Atlantic Forest and known from a few localities in São Paulo and Santa Catarina states. Phylogenetic analyses based on morphological traits and DNA sequences [1143bp of cytochrome *b* (Cyt *b*) and 1235bp of interphotoreceptor retinoid binding protein (IRBP) genes] revealed the placement of *Drymoreomys albimaculatus* in the tribe Oryzomyini, raising to 30 the number of extant Oryzomyini genera. According to those analyses, Percequillo et al. (2011) revealed that *Drymoreomys albimaculatus* is the sister species of the Andean rat *Eremoryzomys polius*.

Here, we describe the karyotype of *Drymoreomys albimaculatus* for the first time. In order to investigate the molecular identification of the two karyotyped animals, we added its Cyt *b* and IRBP sequences to the molecular data published by Percequillo et al. (2011). Additionally, we present morphological comments on the specimens.

## Material and methods

### Sampling

One male and one female were collected with pitfall traps in Santa Virgínia, Parque Estadual da Serra do Mar [45°03.00' to 45°11.00'W (DDM); 23°24.00' to 23°17.00'S (DDM)], state of São Paulo, Brazil. Pelage color and external measurements were taken during the fieldwork. Vouchers of both individuals are deposited in the Coleção de Mamíferos da Universidade Federal do Espírito Santo (UFES) under the catalog numbers UFES 2271 and UFES 2272.

### Cytogenetic analyses

Metaphases were obtained *in vivo* from spleen and bone marrow, according to Ford and Hamerton (1956) with modifications. Conventional Giemsa staining was used to determine the diploid (2n) and the number of autosome arms (FN). GTG and CBG-banding were performed according to Seabright (1971) and Sumner (1972), respectively, with modifications. Fluorescent *in situ* hybridization (FISH) with a FITC labeled (C_3_TA_2_) _n_peptide nucleic acid (PNA) probe (DAKO) was carried out following the recommended protocol (Telomere PNA FISH Kit/FITC, Code No. K5325, DAKO). Mitotic plates were digitally captured with visible light or blue and green filters (emission at 461 and 517 nm, respectively) in an Axioskop 40 epifluorescence microscope (Carl Zeiss) equipped with an Axiocam camera and AxionVision software. Images were overlaid and contrast enhanced with Adobe Photoshop CS5.1.

### DNA extraction, amplification, and sequencing

DNA was extracted from liver with Chelex 5% (Bio-Rad) following Walsh et al. (1991). Amplification of an 820 bp fragment of Cyt *b* and a 782 bp of IRBP was performed with PCR using primers MVZ5 and MVZ16 (Irwin et al. 1991; Smith and Patton 1993), and A1 and F (Stanhope et al. 1992), respectively. Both extraction and PCR controls were used for each amplification. Each PCR mixture had 30 ng of DNA, 25 pmol of each primer, 0.2 mM of dNTP, and 2.52 µL of reaction buffer (50 mM KCl, 2.5 mM MgCl2, 10 mM Tris-HCl; pH 8.8), and 0.2 units of Taq DNA polymerase (Invitrogen) were added to complete 18 µL. Forty amplification cycles were performed in a thermal cycler (Eppendorf Mastercycler ep Gradient, Model 5341). Each cycle consisted of denaturation at 94°C for 30 s, annealing at 48°C for 45 s, and extension at 72°C for 45 s for Cyt *b*, and denaturation at 94°C for 30 s, annealing at 60°C for 60 s, and extension at 72°C for 180 s for IRBP. A final extension at 72°C for 5 min was performed for both Cyt *b* and IRBP amplifications. The PCR products were separated using 1% agarose gels in TAE buffer. Nucleotide sequencing was conducted using BigDye Terminator v3.1 Cycle Sequencing Kit (Applied Biosystems) and an ABI PRISM 3100 Genetic Analyzer (Applied Biosystems). Sequences of each animal were aligned with sequences from previously published data deposited on GenBank by Bonvicino and Moreira (2001), Weksler (2003), and Percequillo et al. (2011) using MAFFT ver. 5 (Katoh et al. 2005) under the iterative method of global pairwise alignment (G-INS-i). Our sequences were submitted to GenBank under accession numbers KF031014-KF031017.

### Phylogenetic analyses

We performed maximum likelihood (ML) and Bayesian analyses using concatenated Cyt *b*-IRBP data set. For both analyses we used gene-specific unlinked models. The best-fitting model of nucleotide substitution for each gene was selected using the Akaike information criterion in accordance with the procedure outlined by Posada and Buckley (2004), and implemented in jModelTest, version 0.1.1 (Posada 2008). The maximum-likelihood trees were calculated using RAxML (Stamatakis 2006). The statistical support for the nodes was estimated by the nonparametric bootstrap, with 1000 pseudoreplicates (Felsenstein 1985). Bayesian analysis was performed using MrBayes 3.04b (Ronquist and Huelsenbeck 2003). Markov chains were started from a random tree and run for 1.0 × 10^7^ generations, sampling every 1000th generation. The stationary phase was checked following Nylander et al. (2004). Sample points prior to the plateau phase were discarded as burn-in, and the remaining trees were combined to find the maximum *a posteriori* probability estimated of the phylogeny. Branch support was estimated by Bayesian posterior probabilities (BPP). Two simultaneous analyses were performed to ensure convergence on topologies.

## Results

### Cytogenetic analyses

**Figure 1.**
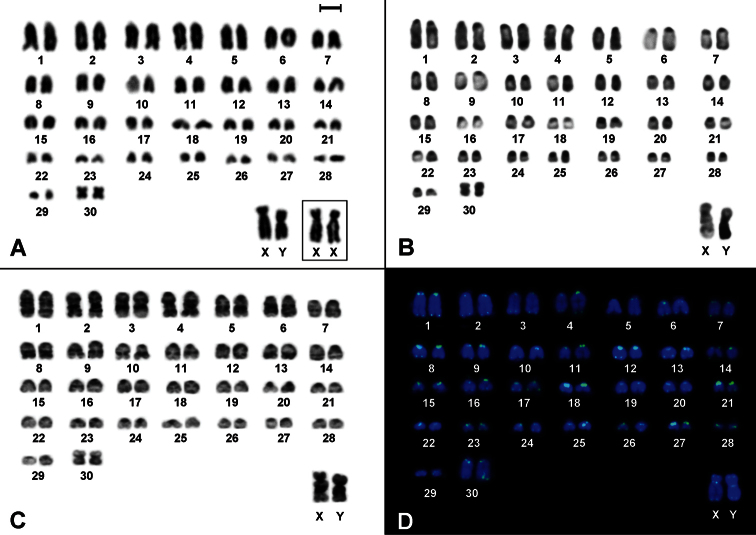
Cytogenetic analyses in *Drymoreomys albimaculatus* from Santa Virgínia, state of São Paulo, Brazil. **A** Karyotype of male (2n=62, FN=62), after conventional staining. Inset: sex chromosomes of a female **B** CBG-banding of a male **C** GTG-banding of a male **D** Fluorescent *in situ* hybridization using telomeric PNA probe over male mitotic plates. Bar scale = 10 μm.

The animals showed 2n=62, FN=62, and the autosome set composed of 29 acrocentric pairs decreasing in size, and one small metacentric pair (Fig. 1A). The X is a large submetacentric, and the Y is a large submetacentric slightly smaller than the X (Fig. 1A). CBG-banding revealed pericentromeric constitutive heterochromatic blocks in all autosomes and in the long arm of Y (Fig. 1B). GTG-banding allowed the identification of almost all autosomic pairs, the X chromosome exhibited two interstitial bands at the long arm while a conspicuous pattern in the Y was not found (Fig. 1C). FISH detected telomeric signals at the ends of all chromosomes and additional telomeric sequences were found in the pericentromeric region of both X and Y chromosomes (Fig. 1D).

### Phylogenetic analyses

**Figure 2.**
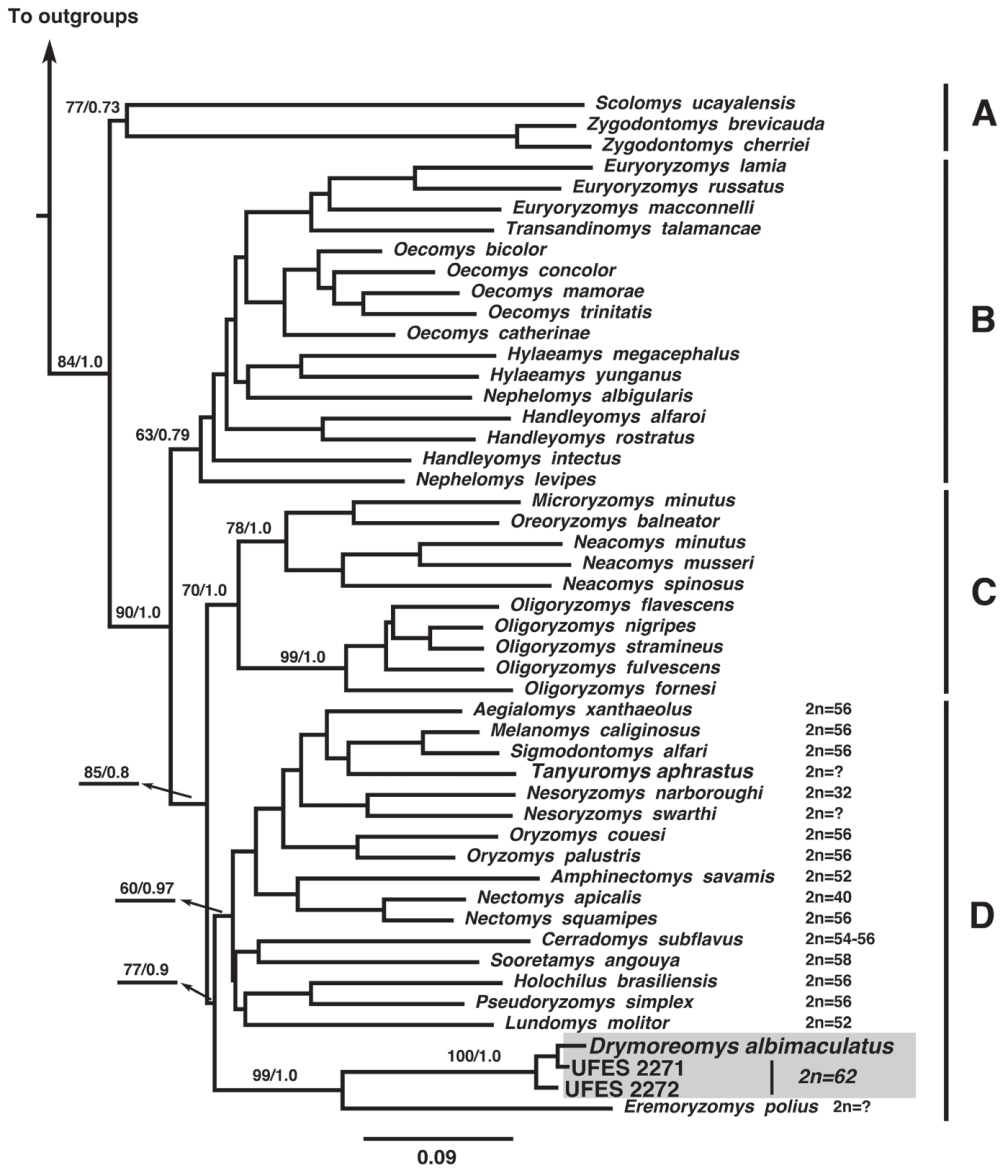
Maximum likelihood tree of combined molecular datasets [cytochrome b (Cyt *b*), interphotoreceptor retinoid binding protein (IRBP)] using Santa Virgínia specimens (UFES2271, UFES2272). Bootstrap nodal support indices and Bayesian posterior probabilities are shown above the branches, respectively. Outgroups include *Peromyscus maniculatus* (Neotominae); *Nyctomys sumichrasti* (Tylomyinae), *Delomys sublineatus* (Sigmodontinae), *Thomasomys baeops* (Sigmodontinae), and *Wiedomys pyrrhorhinos* (Sigmodontinae). Available diploid numbers (2n) of clade D are indicated (for details see Table 1), although the lowest diploid number (*Nectomys palmipes*, 2n= 16, Barros et al. 1992) does not appear in the figure.

The model selected for the phylogenetic analyses (ML and Bayesian) was GTR +I + Γ for each gene. The best ML tree had a -ln likelihood score of -22,345.02. The Bayesian analysis recovered a consensus topology similar to the best ML tree and the results recovered the four well-supported clades A, B, C, and D (Fig. 2) previously reported by Weksler (2006) and Percequillo et al. (2011). In both phylogenetic analyses, Santa Virgínia specimens (UFES 2271 and UFES 2272) clustered with high statistical support to the recently described *Drymoreomys albimaculatus* (Fig. 2, grey area).

## Discussion

Phylogenetic analyses (ML and Bayesian) recovered the four clades A, B, C, and D (Fig. 2) recovered by Weksler (2006) and Percequillo et al. (2011). In both phylogenetic reconstructions, Santa Virgínia specimens were recovered with high statistical support in clade D, confirming their identity as *Drymoreomys albimaculatus* (Fig. 2, grey area), and consistent with Percequillo et al. (2011). Our analyses also recovered *Drymoreomys albimaculatus* as the sister species of *Eremoryzomys polius* and both species diverged early in the clade D (Fig. 2).

The diploid number of *Drymoreomys albimaculatus* corroborates the pattern found for the majority of the Oryzomyini species, in which karyotypes present relatively high chromosome number and predominantly acrocentric pairs. The typical heterochromatic pattern of sex chromosomes is also found in most of the oryzomyine species and it is an essential condition for the recognition of the Y (Fig. 1B).

The karyotype herein reported for *Drymoreomys albimaculatus* is species-specific,since only three other Oryzomyini species present the same diploid number, but different FN: *Oligoryzomys fornesi* (2n=62, FN=64), *Oligoryzomys delicatus* (2n=62, FN=74 and 76), and *Oligoryzomys nigripes* (2n=62, FN=80, 81 and 82) (Gardner and Patton 1976; Weksler and Bonvicino 2005). Telomeric sequences at the pericentromeric region of *Drymoreomys albimaculatus*’ sex chromosomes could be hypothesized as (i) similar to regular sequences of the centromeres, (ii) related to a amplification of (T_2_AG_3_) _n_-like satellite DNA repeats, or (iii) resulted of a structural rearrangement. In fact, interstitial telomeric sequences are common in vertebrates (Meyne et al. 1990) and apparently are a structural component of mammalian satellite DNA (Garagna et al. 1997; Pagnozzi et al. 2000). Additionally, these sequences have been associated with chromosome rearrangements (Ruiz-Herrera et al. 2008; Bolzan 2012).

A compilation of karyological studies in representative species of clade D is presented in Table 1. Notably, cytogenetic data in Oryzomyini has increased considerably in the last decades, mainly because the karyotype has become a valid tool for identifying species of this group. Although several species still remain without karyotypic information (e.g., *Eremoryzomys polius*, *Tanyuromys aphrastus*, *Nesoryzomys swarthy*), the diploid number within clade D varies from 16 in *Nectomys palmipes* (Barros et al. 1992) to 62 in *Drymoreomys albimaculatus*. As *Drymoreomys albimaculatus* exhibited the highest diploid number reported hitherto and diverged early in clade D, karyotype evolution in this clade based on the phylogeny (Fig. 2), apparently exhibits a trend toward a decrease in the diploid number. This hypothesis could imply chromosomal plasticity in low 2n ratios as suggested by Gardner and Patton (1976). In this sense, tandem fusions have perhaps played significant role in clade D, resulting in the lower diploid numbers. Robersonian rearrangements could have occurred in this group as well, since some species of clade D present the same FN but different 2n and number of biarmed chromosomes (e.g., *Drymoreomys albimaculatus* and *Cerradomys subflavus*, Table 1). Non-Robertsonian mechanisms such as pericentric inversions, unequal translocations, or whole-arm heterochromatin addition or deletion could also be invoked in those cases of changes in FN but not in 2n (e.g., *Sigmodontomys alfari*,and *Melanomys caliginous*, Table 1).

Species of clade D present sex and supernumerary chromosomes easily identifiable with classical cytogenetic approaches, and some species exhibit sex chromosomes with polymorphisms/heteromorphisms and interstitial telomeric signals (ITS; Table 1, Fig. 1D). Thus, this clade is an excellent model to study origin, evolution, and chromatin composition of these chromosomes. For instance, a superficial morphological comparison among sex chromosomes from Table 1 could suggest the occurrence of pericentric inversions, or whole-arm heterochromatin additions or deletions.

**Table 1.** Cytogenetic characteristics of Oryzomyini species of clade D, with diploid number (2n), fundamental number (FN), morphologies of autosomal pairs and sex chromosomes, polymorphisms described and references. *Supernumerary chromosomes are not included in autosomal morphologies. A=acrocentric; M=metacentric; SM=submetacentric; ST=subtelocentric; ITS=interstitial telomeric signals; NA=not available.<br/>

**Species**	**2n**	**FN**	Autosomal morphologies*	**Sex chromosome morphologies**	**Cytogenetic characteristics**	**References**
*Aegialomys xanthaeolus*	56	58	25 A<br/> 2 M/SM	X: large A<br/> Y: small A		Gardner and Patton (1976)
*Melanomys caliginosus*	56	58	25 A<br/> 2 M	X: large ST<br/> Y: medium ST		Gardner and Patton (1976)
*Sigmodontomys alfari*	56	54	27 A	X: large A<br/> Y: small A		Gardner and Patton (1976)
*Tanyuromys aphrastus*	NA	NA	NA	NA		
*Nesoryzomys narboroughi*	32	50	5 A<br/> 8 M/SM<br/> 2 ST	X: medium A<br/> Y: small A		Gardner and Patton (1976)
*Nesoryzomys swarthi*	NA	NA	NA	NA		
*Oryzomys couesi*	56	56	26 A<br/> 1 M	X: large SM<br/> Y: medium A/ST	Y heteromorphisms	Haiduk et al. (1979)
*Oryzomys palustris*	56	56	26 A<br/> 1 M	X: large A<br/> Y: minute A		Haiduk et al. (1979); Gardner and Patton (1976)
*Amphinectomys savamis*	52	66	NA	NA		Malygin et al. (1994) apud Musser and Carleton (2005)
*Nectomys apicalis*	42	40	20 A	X and Y: A		Patton et al. (2000)
*Nectomys squamipes*	56–59	56	26 A<br/> 1 M	X: large SM/ ST<br/> Y: medium/ small SM/ST	0-2 B chromosomes; sex chromosomes polymorphisms	Maia et al. (1984)
*Cerradomys subflavus*	54–56	62	21 A, 3 SM, 2M<br/> 23 A, 2 SM, 2M	X: large A/ ST<br/> Y: medium A/ large A	Centric fusion/fission, pericentric inversion, sex chromosomes polymorphisms	Almeida and Yonenaga-Yassuda (1985)
*Sooretamys angouya*	58, 60	60, 64	26 A<br/> 2 M	X: large A<br/> Y: medium ST	0 or 2 B chromosomes	Andrades-Miranda et al. (2001); Silva and Yonenaga-Yassuda (2004)
*Holochilus brasiliensis*	56–58	56, 58, 60	26 A<br/> 1 M	X: large ST<br/> Y: small SM	0 to 2 B chromosomes	Yonenaga-Yassuda et al. (1987)
*Pseudoryzomys simplex*	56	54, 55	27 A	X: large A<br/> Y: medium A	Heteromorphic pair 17 due to addition of constitutive heterochromatin	Voss and Myers (1991); Moreira et al. (in press)
*Lundomys molitor*	52	58	21 A<br/> 4 M	X: large SM<br/> Y: small M	X heteromorphism	Freitas et al. (1983)
*Drymoreomys albimaculatus*	62	62	29 A<br/> 1M	X: large SM<br/> Y: medium SM	ITS in both sex chromosomes	Present study
*Eremoryzomys polius*	NA	NA	NA	NA	NA	

### Comments on external morphology and natural history

The specimens collected were medium sized (male body mass: 46.5 g, head and body length: 115 mm, and tail length: 142 mm; female body mass: 57 g, head and body length: 127 mm, and tail length: 170 mm). Tail was longer than head and body, and was a uniform color on both sides. Male hind footwas short (25 mm, 22% of head and body length) and ears were small (16 mm; 14% of head and body length). These external morphological measures overlapped with those of the *Drymoreomys albimaculatus* holotype (Percequillo et al. 2011). Dorsal pelage was reddish-brown; ventral pelage was predominantly grayish. Samples exhibited the pattern of short hind feet consistent with *Oecomys*. Fore and hind feet digits were covered by silvery-white hairs and the dorsal surface of hind feet were covered by brown hairs forming a patch, in a more conspicuous pattern than the observed for *Rhipidomys*. Thus, some external morphological traits were similar to those described for *Rhipidomys* and *Oecomys* as reported Percequillo et al. (2011). Nevertheless, our samples exhibited the characteristics of the *Drymoreomys albimaculatus* holotype that differentiate it from *Rhipidomys*, such as the shorter, thinner, and sparser mystacial vibrissae and presence of gular to pectoral patches of white hair. Additionally, we detected that, contrary to what is found in *Rhipidomys*, a tuft of hairs on the tail’s end is absent in our samples. On the other hand, several anatomical traits that distinguish the *Drymoreomys albimaculatus* holotype and species of *Oecomys* wereobserved in our samples, such as the plantar surface of pes covered with squamae; dorsal surface of pes with dark patches of brown hairs and the ventral pelage with gular and thoracic white patches (Percequillo et al. 2011).

Percequillo et al. (2011) reported that most of the *Drymoreomys* specimens were collected in pitfall traps; in the present work, the animals were also collected in the same way. These reiterate the importance of further fieldwork effort, with different collecting methods in order to increase the spectrum of small mammals collected. Consequently, our knowledge of small mammal biodiversity will be improved as a whole, which will allow improvements in relevant laws and policies for biodiversity protection.

## References

[B1] Almeida EJC Yonenaga-Yassuda Y (1985) Robertsonian fusion, pericentric inversion and sex-chromosome heteromorphisms in *Oryzomys subflavus* (Cricetidae, Rodentia). Caryologia 38: 129-137.

[B2] Andrades-Miranda J Zanchin NIT Oliveira LFB Langguth AR Mattevi MS (2001) Cytogenetic studies in nine taxa of the genus *Oryzomys* (Rodentia, Sigmodontinae) from Brazil. Mammalia 65: 461-472. doi: 10.1515/Mamm.2001.65.4.461

[B3] Barros MA Reig OA Perez-Zapata A (1992) Cytogenetics and karyosystematics of South American Oryzomyine rodents (Cricetidae, Sigmodontinae). Cytogenetics and Cell Genetics 59: 34-38. doi: 10.1159/000133195 1733670

[B4] Bolzan AD (2012) Chromosomal aberrations involving telomeres and interstitial telomeric sequences. Mutagenesis 27: 1-15. 21857006 10.1093/mutage/ger052

[B5] Bonvicino CR Moreira MA (2001) Molecular phylogeny of the genus *Oryzomys* (Rodentia: Sigmodontinae) based on cytochrome *b* DNA sequences. Molecular Phylogenetics and Evolution 18: 282-292. doi: 10.1006/mpev.2000.0878 11161762

[B6] Christoff AU Fagundes V Sbalqueiro IJ Mattevi MS Yonenaga-Yassuda Y (2000) Description of a new species of *Akodon* (Rodentia: Sigmodontinae) from southern Brazil. Journal of Mammalogy 81: 838-851. doi: 10.1644/1545-1542(2000)081<0838:Doanso>2.3.Co;2

[B7] Costa BMDA Geise L Pereira LG Costa LP (2011) Phylogeography of *Rhipidomys* (Rodentia: Cricetidae: Sigmodontinae) and description of two new species from southeastern Brazil. Journal of Mammalogy 92: 945-962. doi: 10.1644/10-mamm-a-249.1

[B8] Costa LP Pavan SE Leite YLR Fagundes V (2007) A new species of *Juliomys* (Mammalia: Rodentia: Cricetidae) from the Atlantic forest of southeastern Brazil. Zootaxa 1463: 21-37. doi: 10.2307/2806242

[B9] De Vivo M Carmignotto AP Gregorin R Hingst-Zaher E Lack-Ximenes GE Miretzki M Percequillo AR Rollo-Jr MM Rossi RV Taddei VA (2010) Checklist of mammals from São Paulo State, Brazil. Biota Neotropica http://www.biotaneotropica.org.br/v11n1a/pt/abstract?inventory+bn0071101a2011:

[B10] Felsenstein J (1985) Confidence limits on phylogenies: an approach using the bootstrap. Evolution 39: 783-791. doi: 10.2307/2408678 28561359

[B11] Ford CE Hamerton JL (1956) A colchicine, hypotonic citrate, squash sequence for mammalian chromosomes. Stain Technology 31: 247-251. doi: 10.3109/10520295609113814 13380616

[B12] Freitas TRO Mattevi MS Oliveira LFB Souza MJ Yonenaga-Yassuda Y Salzano FM (1983) Chromosome relationships in three representatives of the genus *Holochilus* (Rodentia, Cricetidae) from Brazil. Genetica 61: 13-20. doi: 10.1007/bf00563228

[B13] Garagna S Ronchetti E Mascheretti S Crovella S Formenti D Rumpler Y Manfredi Romanini MG (1997) Non-telomeric chromosome localization of (TTAGGG)_n_ repeats in the genus *Eulemur*. Chromosome Research 5: 487-491. doi: 10.1023/A:1018425215516 9421267

[B14] Gardner AL Patton JL (1976) Karyotypic variation in Oryzomyine Rodents (Cricetinae) with comments on chromosomal evolution in the neotropical Cricetine Complex. Occasional Papers of the Museum of Zoology, Louisiana State University 49: 1-47.

[B15] González EM Langguth A Oliveira LF (1999) A new species of *Akodon* from Uruguay and southern Brazil: (Mammalia: Rodentia: Sigmodontinae). Comunicaciones Zoológicas del Museo de Historia Natural de Montevideo 191: 1-8.

[B16] Haiduk MW Bickham JW Schmidly DJ (1979) Karyotypes of six species of *Oryzomys* from Mexico and Central America. Journal of Mammalogy 60: 610-615. doi: 10.2307/1380103

[B17] Irwin DM Kocher TD Wilson AC (1991) Evolution of the cytochrome *b* gene of mammals. Journal of Molecular Evolution 32: 128-144. doi: 10.1007/BF02515385 1901092

[B18] Katoh K Kuma K Toh H Miyata T (2005) MAFFT version 5: improvement in accuracy of multiple sequence alignment. Nucleic Acids Research 33: 511-518. doi: 10.1093/nar/gki198 15661851 PMC548345

[B19] Maia V Yonenaga-Yassuda Y Freitas TRO Kasahara S Sune-Mattevi M Oliveira LF Galindo MA Sbalqueiro IJ (1984) Supernumerary chromosomes, robertsonian rearrangement and variability of the sex-chromosomes in *Nectomys squamipes* (Cricetidae, Rodentia). Genetica 63: 121-128. doi: 10.1007/Bf00605896

[B20] Malygin VM Aniskin VM Isaev SI Milishnikov AN (1994) *Amphinectomys savamis* Malygin Gen-N Et Sp-N, a new genus and a new species of water rat (Cricetidae, Rodentia) from Peruvian Amazonia. Zoologichesky Zhurnal 73: 195-208.

[B21] Mares MA Braun JK (2000) Three new species of *Brucepattersonius* (Rodentia: Sigmodontinae) from Misiones Province, Argentina. Occasional Papers of the Sam Noble Oklahoma Museum of Natural History 9: 1-3.

[B22] Meyne J Baker RJ Hobart HH Hsu TC Ryder OA Ward OG Wiley JE Wurster-Hill DH Yates TL Moyzis RK (1990) Distribution of non-telomeric sites of the (TTAGGG)_n_telomeric sequence in vertebrate chromosomes. Chromosoma 99: 3-10. doi: 10.1007/BF01737283 2340757

[B23] Moreira CN Di-Nizo CB Silva MJJ Yonenaga-Yassuda Y Ventura K (in press) A remarkable autosomal heteromorphism in *Pseudoryzomys simplex* 2n=56, NFa=54, 55 (Rodentia, Sigmodontinae). Genetics and Molecular Biology. 10.1590/S1415-47572013000200010 PMC3715286 23885202

[B24] Musser GG Carleton MD (2005) Superfamily Muroidea. In: Wilson DE Reeder DM (Eds). Mammal Species of the World: A Taxonomic and Geographic Reference. John Hopkins University Press, Baltimore: 894-1531.

[B25] Nylander JA Ronquist F Huelsenbeck JP Nieves-Aldrey JL (2004) Bayesian phylogenetic analysis of combined data. Systematic Biology 53: 47-67. doi: 10.1080/10635150490264699 14965900

[B26] Oliveira JA Bonvicino CR (2002) A new species of sigmodontine rodent from the Atlantic forest of eastern Brazil. Acta Theriologica 47: 307-322. doi: 10.1007/BF03194149

[B27] Pagnozzi JM Silva MJJ Yonenaga-Yassuda Y (2000) Intraspecific variation in the distribution of the interstitial telomeric (TTAGGG)_n_ sequences in *Micoureus demerarae* (Marsupialia: Didelphidae). Chromosome Research 8: 585-591. doi: 10.1023/A:1009229806649 11117354

[B28] Pardiñas UFJ D’Elia G Cirignoli S Suarez P (2005) A new species of *Akodon* (Rodentia, Cricetidae) from the northern campos grasslands of Argentina. Journal of Mammalogy 86: 462–474. doi: 10.1644/1545-1542(2005)86[462:Ansoar]2.0.Co;2

[B29] Pardiñas UFJ Teta P D’Elia G (2009) Taxonomy and distribution of *Abrawayaomys* (Rodentia: Cricetidae), an Atlantic Forest endemic with the description of a new species. Zootaxa: 39–60. doi: 10.11646/zootaxa.3641.4.9

[B30] Patton JL Da Silva MNF Malcolm JR (2000) Mammals of the Rio Juruá and the evolutionary and ecological diversification of Amazonia. Bulletin of the American Museum of Natural History 244: 1-306. doi: 10.1206/0003-0090(2000)244<0001:motrja>2.0.co;2

[B31] Percequillo AR Hingst-Zaher E Bonvicino CR (2008) Systematic review of genus *Cerradomys* Weksler, Percequillo and Voss, 2006 (Rodentia: Cricetidae: Sigmodontinae: Oryzomyini), with description of two new species from eastern Brazil. American Museum Novitates: 1–46. doi: 10.1206/495.1

[B32] Percequillo AR Weksler M Costa LP (2011) A new genus and species of rodent from the Brazilian Atlantic Forest (Rodentia: Cricetidae: Sigmodontinae: Oryzomyini), with comments on oryzomyine biogeography. Zoological Journal of Linnean Society 161: 357-390. doi: 10.1111/j.1096-3642.2010.00643.x

[B33] Posada D (2008) jModelTest: phylogenetic model averaging. Molecular Biology and Evolution 25: 1253-1256. doi: 10.1093/molbev/msn083 18397919

[B34] Posada D Buckley TR (2004) Model selection and model averaging in phylogenetics: advantages of akaike information criterion and bayesian approaches over likelihood ratio tests. Systematic Biology 53: 793-808. doi: 10.1080/10635150490522304 15545256

[B35] Ribeiro MC Metzger JP Martensen AC Ponzoni FJ Hirota MM (2009) The Brazilian Atlantic Forest: how much is left, and how is the remaining forest distributed? Implications for conservation. Biological Conservation 142: 1141-1153. doi: 10.1016/J.Biocon.2009.02.021

[B36] Ronquist F Huelsenbeck JP (2003) MrBayes 3: Bayesian phylogenetic inference under mixed models. Bioinformatics 19: 1572-1574. doi: 10.1093/bioinformatics/btg180 12912839

[B37] Ruiz-Herrera A Nergadze SG Santagostino M Giulotto E (2008) Telomeric repeats far from the ends: mechanisms of origin and role in evolution. Cytogenetic and Genome Research 122: 219-228. doi: 10.1159/000167807000167807 19188690

[B38] Seabright M (1971) A rapid banding technique for human chromosomes. Lancet 2: 971-972. doi: 10.1016/S0140-6736(71)90287-X 4107917

[B39] Silva MJJ Yonenaga-Yassuda Y (2004) B chromosomes in Brazilian rodents. Cytogenetic and Genome Research 106: 257-263. doi: 10.1159/000079296 15292600

[B40] Smith MF Patton JL (1993) The diversification of South American murid rodents: evidence from mitochondrial DNA sequence data for the Akodontine tribe. Biological Journal of the Linnean Society 50: 149-177. doi: 10.1111/J.1095-8312.1993.Tb00924.X

[B41] Stamatakis A (2006) RAxML-VI-HPC: maximum likelihood-based phylogenetic analyses with thousands of taxa and mixed models. Bioinformatics 22: 2688-2690. doi: 10.1093/bioinformatics/btl446 16928733

[B42] Stanhope MJ Czelusniak J Si JS Nickerson J Goodman M (1992) A molecular perspective on mammalian evolution from the gene encoding interphotoreceptor retinoid binding protein, with convincing evidence for bat monophyly. Molecular Phylogenetics and Evolution 1: 148-160. doi: 10.1016/1055-7903(92)90026-D 1342928

[B43] Sumner AT (1972) A simple technique for demonstrating centromeric heterochromatin. Experimental Cell Research 75: 304-306. 4117921 10.1016/0014-4827(72)90558-7

[B44] Voss RS Myers P (1991) *Pseudoryzomys simplex* (Rodentia, Muridae) and the significance of lund collections from the caves of Lagoa-Santa, Brazil. Bulletin of the American Museum of Natural History: 414–432.

[B45] Walsh PF Metzger DA Higuchi R (1991) Chelex 100 as a medium for simple extraction of DNA for PCR-based typing from forensic material. Biotechniques 10: 506-513. 1867860

[B46] Weksler M (2003) Phylogeny of Neotropical oryzomyine rodents (Muridae: Sigmodontinae) based on the nuclear IRBP exon. Molecular Phylogenetics and Evolution 29: 331-349. 13678688 10.1016/s1055-7903(03)00132-5

[B47] Weksler M (2006) Phylogenetic relationships of oryzomine rodents (Muroidea: Sigmodontinae): separate and combined analyses of morphological and molecular data. Bulletin of the American Museum of Natural History 296: 1-48.

[B48] Weksler M Bonvicino CR (2005) Taxonomy of pigmy rice rats genus *Oligoryzomys* Bangs, 1900 (Rodentia, Sigmodontinae) of the Brazilian Cerrado, with the description of two new species. Arquivos do Museu Nacional Rio de Janeiro 63: 113-130.

[B49] Weksler M Geise L Cerqueira R (1999) A new species of *Oryzomys* (Rodentia, Sigmondontinae) from southeast Brazil, with comments on the classification of the *O. capito* species group. Zoological Journal of the Linnean Society 125: 445-462. doi: 10.1111/J.1096-3642.1999.Tb00600.X

[B50] Yonenaga-Yassuda Y Prado RCD Mello DA (1987) Supernumerary chromosomes in *Holochilus brasiliensis* and comparative cytogenetic analysis with *Nectomys squamipes* (Cricetidae, Rodentia). Revista Brasileira de Genetica 10: 209-220.

